# Heterozygosity for Nuclear Factor One X Affects Hippocampal-Dependent Behaviour in Mice

**DOI:** 10.1371/journal.pone.0065478

**Published:** 2013-06-11

**Authors:** Lachlan Harris, Chantelle Dixon, Kathleen Cato, Yee Hsieh Evelyn Heng, Nyoman D. Kurniawan, Jeremy F. P. Ullmann, Andrew L. Janke, Richard M. Gronostajski, Linda J. Richards, Thomas H. J. Burne, Michael Piper

**Affiliations:** 1 The School of Biomedical Sciences, The University of Queensland, Brisbane, Australia; 2 The Queensland Brain Institute, The University of Queensland, Brisbane, Australia; 3 Centre for Advanced Imaging, The University of Queensland, Brisbane, Australia; 4 Department of Biochemistry and the Program in Neuroscience, Developmental Genomics Group, New York State Center of Excellence in Bioinformatics and Life Sciences, State University of New York at Buffalo, Buffalo, New York, United States of America; University of South Florida, United States of America

## Abstract

Identification of the genes that regulate the development and subsequent functioning of the hippocampus is pivotal to understanding the role of this cortical structure in learning and memory. One group of genes that has been shown to be critical for the early development of the hippocampus is the *Nuclear factor one* (*Nfi*) family, which encodes four site-specific transcription factors, NFIA, NFIB, NFIC and NFIX. In mice lacking *Nfia, Nfib* or *Nfix*, aspects of early hippocampal development, including neurogenesis within the dentate gyrus, are delayed. However, due to the perinatal lethality of these mice, it is not clear whether this hippocampal phenotype persists to adulthood and affects hippocampal-dependent behaviour. To address this we examined the hippocampal phenotype of mice heterozygous for *Nfix* (*Nfix* ^+/−^), which survive to adulthood. We found that *Nfix* ^+/−^ mice had reduced expression of NFIX throughout the brain, including the hippocampus, and that early hippocampal development in these mice was disrupted, producing a phenotype intermediate to that of wild-type mice and *Nfix^−/−^* mice. The abnormal hippocampal morphology of *Nfix* ^+/−^ mice persisted to adulthood, and these mice displayed a specific performance deficit in the Morris water maze learning and memory task. These findings demonstrate that the level of *Nfix* expression during development and within the adult is essential for the function of the hippocampus during learning and memory.

## Introduction

The prevalence of neurodevelopmental disorders associated with malformation of the hippocampus, such as epilepsy [1] and schizophrenia [2], highlights the importance of understanding the events underpinning hippocampal development. Altman and Bayer first elucidated the fundamental principles of hippocampal development over two decades ago [3], [4], demonstrating that the neuronal layers of the hippocampus, the dentate gyrus and the cornu ammonis (CA) regions, arise from distinct zones within the hippocampal neuroepithelium from approximately embryonic day (E) 14 in rodents. However, our understanding of the genes controlling hippocampal development still remains comparatively limited.

Recent studies have demonstrated that Nuclear factor one (NFI) transcription factors, which have previously been implicated in the development of the spinal cord [5], neurogenesis within the cerebellum [6], [7] and corpus callosum formation [8], [9], are also critical regulators of the embryonic development of the hippocampus [10]–[12]. During this period, NFIA, NFIB and NFIX are all highly expressed by progenitor cells in the hippocampal neuroepithelium, and by post-mitotic neurons and glia within the hippocampal anlage [13], [14]. In *Nfix* knockout mice the early morphological development of the hippocampus is severely disrupted, with progenitor cells within the hippocampal neuroepithelium failing to differentiate according to normal developmental timelines. This results in delayed glial development and deficits with respect to the production of glutamatergic neurons of the CA regions of the hippocampus and of granule neurons in the dentate gyrus, defects that ultimately culminate in the severe morphological reduction of the hippocampal dentate gyrus and hippocampal fissure at E18 [11]. NFI proteins are postulated to regulate the embryonic development of the hippocampus, as well as other regions of the nervous system, via two complementary mechanisms. Firstly, NFI transcription factors have been shown to promote the differentiation of neural progenitor cells into post-mitotic neurons and glia by negatively regulating genes that maintain progenitor cell identity, such as *Hes1* and *Sox9* [11], [12]. Secondly, they have also been shown to promote neuronal differentiation by the activation of neuronal-specific genes such as *Tag-1* and *Wnt7a* [15], and glial differentiation by activating the expression of glial-specific genes such as *Gfap* and *B-fapb* [16], [17].

While disrupting *Nfi* gene expression affects the embryonic development of the hippocampus, it is unclear what effect this has on hippocampal-dependent behaviour in the adult nervous system. The majority of *Nfia* knockout mice (>95%) and all *Nfib* knockout mice die at birth, precluding investigation of these mice at later developmental time-points. Similarly, *Nfix* knockout mice maintained on a C57Bl/6J background only survive until postnatal day (P) 21, making the contribution of this gene to hippocampal function in the adult brain impossible to assess with this particular homozygous line [11]. However, mice heterozygous for *Nfix* have normal viability, making them an ideal model to investigate whether disrupting the normal level of *Nfix* expression during development affects the functioning of the hippocampus within adult mice. Here, we reveal that *Nfix* ^+/−^ mice display reduced expression of NFIX and that early hippocampal development is delayed in these mice, producing a phenotype intermediate to that of *Nfix* ^+/+^ and *Nfix* ^−/−^ mice. This early developmental phenotype of *Nfix* ^+/−^ mice culminates in aberrant morphology and reduced rates of neurogenesis within the dentate gyrus of the adult hippocampus. Finally, we demonstrate that adult *Nfix* ^+/−^ mice exhibit deficits in a hippocampal-specific learning and memory task. Taken together, these data highlight the importance of NFIX for the normal formation and function of the hippocampus and may provide insights into the underlying basis of the neurological symptoms associated with disorders such as Sotos syndrome, in which mutations in *NFIX* have been implicated [18]–[20].

## Materials and Methods

### Mice

Embryonic, postnatal and adult *Nfix^+/+^* (wild-type) and *Nfix^+/−^* littermate mice, maintained on a C57Bl/6J background, were used for this study. These mice were bred by crossing heterozygous sires with heterozygous or wild-type dams. To determine the age of embryos, female mice were placed with male mice overnight and the following day was designated as E0 if the female had a vaginal plug. Mice older than P60 are referred to as adults in this study. All animals were genotyped using the polymerase chain reaction (PCR) as described previously [13]. All experimental protocols were performed with the approval of The University of Queensland Animal Ethics Committee in accordance with the Australian Code of Practice for the Care and Use of Animals for Scientific Purposes.

### Tissue Preparation and Sectioning

E14 embryos were drop-fixed in 4% (w/v) paraformaldehyde (PFA) solution, whereas older embryos and postnatal animals were transcardially perfused with 0.9% saline followed by 4% PFA. After post-fixing in 4% PFA at 4°C for at least 16 hours, brains were removed from the skull, embedded in 3% noble agar (BD Biosciences) and sectioned coronally at 50 µm using a vibratome (Leica).

### Haematoxylin Staining

Coronal sections were mounted and dried on Superfrost Plus slides before being incubated in Mayer’s haematoxylin (Sigma-Aldrich) solution for 3 minutes. The sections were then washed for 2 minutes with tap water, before being dehydrated in an ethanol-xylene series and cover-slipped using DPX mounting medium (Ajax Finechem). Sections from comparable positions along the rostral-caudal axis of the hippocampus were then imaged using an upright microscope (Zeiss upright Axio-Imager Z1) connected to an Axiocam HRc digital camera.

### Immunohistochemistry

Immunohistochemistry using the chromogen 3,3′-diaminobenzidine (DAB) was performed as described previously [21]. Briefly, floating tissue sections were blocked for 2 hours with normal donkey or goat serum (Jackson Immunoresearch) before being incubated with primary antibody in the same blocking solution overnight. Primary antibodies used for immunohistochemistry were anti-NFIX (rabbit polyclonal, 1/1,000; Abcam), anti-glial fibrillary acidic protein (GFAP) (rabbit polyclonal, 1/10,000; Dako), anti-glutamate aspartate transporter (GLAST) (rabbit polyclonal, 1/50,000; a gift from Dr Niels Danbolt, University of Oslo), anti-doublecortin (DCX) (rabbit polyclonal, 1/50,000; Abcam), anti-prospero-related-homeobox 1 (PROX1) (rabbit polyclonal, 1/100,000; Millipore Bioscience Research Reagents) and anti-paired box 6 (PAX6, rabbit polyclonal, 1/20,000; Millipore Bioscience Research). Sections were then incubated with biotin-conjugated goat anti-rabbit IgG (1/1,000, Vector Laboratories) or donkey anti-mouse IgG (1/1,000, Jackson Immunoresearch) secondary antibodies for 1 hour. The reaction was visualised by incubating the sections in avidin-biotin complex (ABC elite kit; Vector Laboratories) for 1 hour, followed by a nickel-DAB solution, and was terminated by washing multiple times in phosphate buffered saline when a dark precipitate had formed. Sections were then mounted onto slides, dehydrated and imaged as described above. For each immunohistochemical experiment, at least 3 wild-type and 3 *Nfix* ^+/−^ mice were analysed.

### Quantification of Dentate Gyrus Area/cell Counts

All cell counts and area measurements were performed using the free-ware program ImageJ, and were performed with the researcher blinded to the genotype of the samples. To measure the area of the emerging dentate gyrus in E18 wild-type and *Nfix* ^+/−^ mice, tissue sections were stained for PROX1 and imaged at equivalent points along the rostral-caudal axis of the hippocampus. In order to calculate the density of PROX1-positive cells within the dentate gyrus at E18, the number of PROX1-positive cells was counted and divided by the total area of the dentate gyrus. In adult mice, the average width of the suprapyramidal and infrapyramidal blades of the dentate gyrus was quantified by taking measurements at 4 points along the proximal extent of each blade in PROX1- or haematoxylin-stained sections. To quantify the number of type 1 radial progenitors within the dentate gyrus, only cells with a single GFAP-positive process, oriented perpendicular to the granule cell layer and emerging from the subgranular zone (SGZ), were counted. Newly born DCX-positive neurons were defined as immunopositive cells residing within the SGZ or the granule cell layer of the dentate gyrus. For each stain, sections were imaged at equivalent positions along the rostral-caudal axis of the hippocampus, and for each animal both the left and right hippocampi were analysed and averaged. Data for all experiments requiring quantification represent pooled results from at least 5 wild-type and 5 *Nfix* ^+/−^ mice.

### Magnetic Resonance Imaging Analysis

Adult mice were transcardially perfused with 0.9% saline and then fixed with 4% PFA containing 0.1% Magnevist® (gadopentetate dimeglumine; Bayer Schering Pharma AG) in PBS to reduce T1 and enhance magnetic resonance (MR) contrast. The head was then detached and post-fixed overnight in 30 ml of 4% PFA containing 0.1% Magnevist®, after which the brain was dissected from the skull and incubated in 30 ml of PBS containing 0.1% Magnevist® for 4 days, then placed in Fomblin (Solvay Solexis) and imaged on a 16.4T (89 mm) Bruker microimaging system (Bruker Biospin) using a 15 mm SAW coil (M2M Imaging). MR imaging data were acquired using a T1/T2*-weighted 3D gradient echo imaging sequence with the following parameters: repetition time = 50 ms, echo time = 12 ms, flip angle = 30°, 40 KHz spectral bandwidth and 2 excitations, with a total acquisition time of 1 hour 43 minutes. The imaging field-of-view was 1.9×1.2×0.9 cm and the imaging matrix size was 633×400×300, this results in a 30 µm isotropic image resolution. Volumes of hippocampal structures were calculated by non-linear registration to a 15 µm average mouse model in Waxholm space from 16.4T 30 µm images. Model-based segmentation was then performed using the hippocampal regions published by Richards et al., 2011 [22].

### Behavioural Tests

Behavioural tests were performed on 9 to 11 week old wild-type mice (9 male, 11 female) and *Nfix* ^+/−^ mice (12 male, 14 female). After weaning at P21, the mice used in the behavioural tests were housed in same sex groups (2–4 mice per group) in individually ventilated OptiMice cages (Animal Care Systems) with *ad libitum* access to standard mouse food and water. The holding room was kept at a constant temperature of 21±1°C, on a 12 hour light/dark cycle with lights on at 0600. Mice were not handled prior to commencing behavioural tests. All behavioural tests were performed between the hours of 0800–1400, with mice being given 30 minutes to acclimatise to the testing room before each experiment. All data were acquired using EthoVision XT software (Noldus) and experiments were performed blind to the genotype of the mice.

### SHIRPA Screen

A modified SHIRPA screen (SmithKline Beecham, Harwell, Imperial College, Royal London Hospital, phenotype assessment) was used as a first-line phenotyping screen of *Nfix* ^+/−^ mice to measure gross neurological functioning, including assessments of muscular, spinocerebellar, sensory, neuropsychiatric and autonomic functions [23]. The SHIRPA screen took approximately 3 minutes per mouse to complete.

### Open-field Test

The open-field apparatus used in this study was a 30×30×30 cm square white box, illuminated by white light at an intensity level of 35 lux. At the beginning of the test each mouse was placed in the arena facing a corner of the box. Ethovision software was used to record the distance each mouse travelled over a 10 minute test period. Other parameters, including the time each mouse spent in the centre of the box relative to the perimeter of the box, were also recorded.

### Elevated Plus Maze

The elevated plus maze is a widely used test of anxiety-related behaviour in rodents, and consists of an elevated platform with closed and open arms. In this test, wild-type mice spend only a small amount of time in the open arm due to their unconditioned fear of heights and their fear of exposure to predators, such that increased open arm activity reflects anti-anxiety behaviour [24]. The elevated plus maze apparatus was made of opaque grey acrylic and comprised two open arms (30×5 cm) and two closed arms (30×5 cm) that extended from a central platform (5×5 cm) 65 cm above the floor. The light intensity in both the open arms and closed arms was approximately 10 lux. At the beginning of the test each mouse was placed at the centre of the maze facing one of the open arms, after which its activity was recorded for 10 minutes. Ethovision software recorded the amount of time each mouse spent in the open arms, closed arms and centre of the maze, as well as the frequency of open arm entries.

### Morris Water Maze

The Morris water maze test used in this study took place over 10 days. The test arena had a diameter of 80 cm and a depth of 40 cm. For the duration of the test period the apparatus was filled with water coloured by non-toxic white paint that was maintained at a temperature of 25±1°C. Mice underwent pre-training a day before testing began, during which they were trained to swim to a visible platform (5 cm diameter; marked by a flag) located at the centre of the maze. This pre-training, which took place over two trials and ended when the mouse found the platform, was conducted so that mice would begin to associate the platform with escape from the maze, thereby reducing instances of floating caused by learned helplessness [25].

On the first 5 days (days 1–5) of the test, the platform was located in the north-east quadrant of the test arena and was hidden beneath 1.5 cm of water. On each of these 5 days mice were given 4 trials, lasting 1 minute each, in which to locate the hidden platform (inter-trial period of 4 to 6 minutes). At the start of each trial the mouse was released into the test arena from one of four starting positions chosen in a pseudo-randomised order. If the mouse was unable to locate the platform within 1 minute it was guided to the platform until it remained there for 10 seconds, before being towel dried and returned to its home cage. The average latency for each mouse to find the platform over the four trials for each testing day was then recorded. On day 6 of the test a single probe trial was performed. In this probe trial the platform was removed from the arena and each mouse was allowed to search for the platform for 1 minute. The time spent in the target quadrant and the average search proximity, measured as the mean distance from the platform in its trained position over the 1 minute trial, was recorded for each mouse. On days 7 and 8 a reversal learning procedure was performed, where the platform was re-introduced into the test arena in a new position within the south-west quadrant. Again, mice were trained to find the platform over 4 trials for each testing day. On day 9 another probe trial was performed where the platform was again removed from the arena. In this probe trial the time spent in the target quadrant and the search proximity of each mouse to the platform in its trained position in the south-west quadrant and its previously trained position (north-east quadrant; days 1–5) were recorded.

### Statistical Analyses

Quantitative data were analysed using SPSS statistical software (version 20, IBM) to determine significance. Two-tailed unpaired Students *t*-tests were performed when comparing two groups; *p* values from Students *t*-tests are reported in the text with *t* statistic and degrees of freedom. For experiments involving two independent variables two-way ANOVA was performed, with repeated-measures if applicable; *p* values from two-way ANOVA are reported in the text with the *F*-statistic and the between-subjects and within-subjects degrees of freedom. Any significant main effect of genotype detected by two-way ANOVA was followed by multiple *t*-tests using a pooled estimate of variance where appropriate. The family-wise error rate was then adjusted using the Bonferonni correction. As we found no significant main effect of gender for the behavioural experiments conducted in this study, all data are presented pooled for sex. Error bars represent the standard error of the mean.

## Results

### *Nfix^+/−^* mice have Reduced Hippocampal Expression of NFIX during Development and in Adulthood

**Figure 1:**
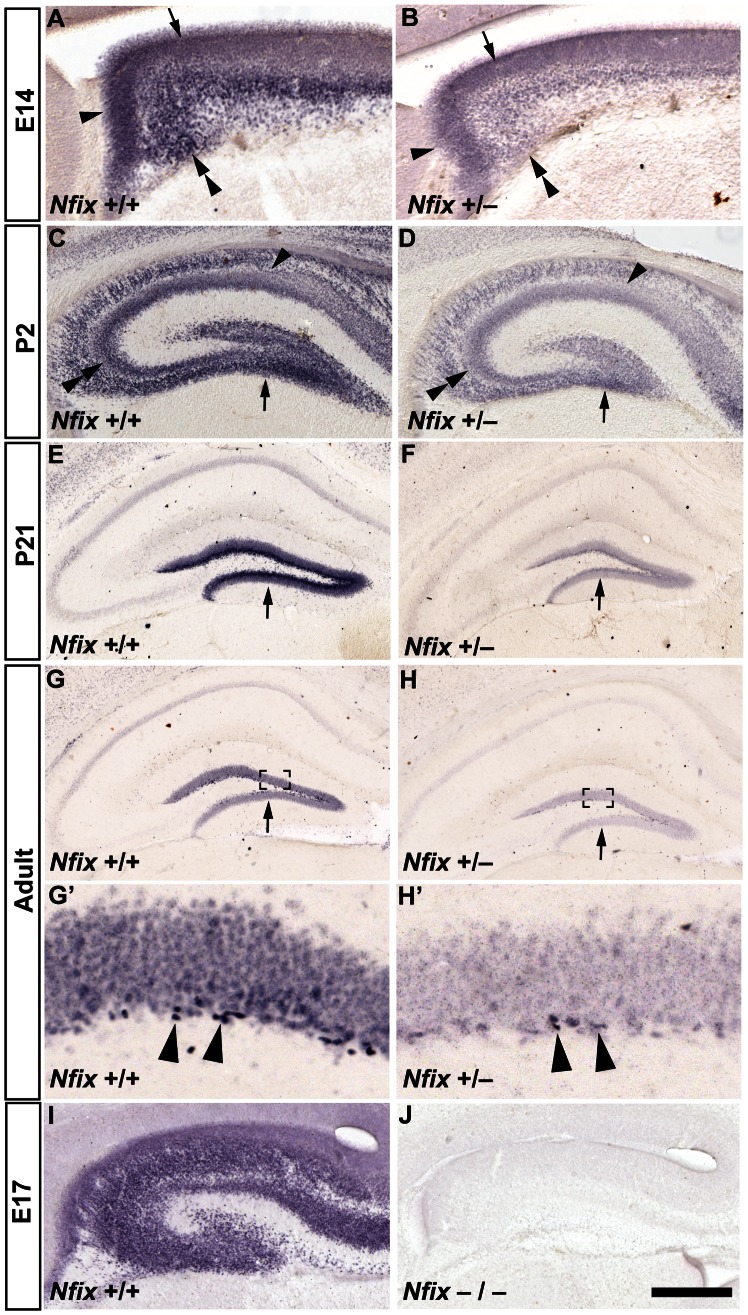
*Nfix^+/−^* mice have reduced hippocampal expression of NFIX. Anti-NFIX staining of wild-type and *Nfix* ^+/−^ coronal brain sections at embryonic, postnatal and adult ages (**A–H’**). In E14 wild-type mice (**A**) NFIX was detected within two regions of the hippocampal ventricular zone, the ammonic neuroepithelium (arrow in **A**) and the dentate neuroepithelium (arrowhead in **A**), as well as by post-mitotic neurons within the developing hippocampal anlage (double arrowhead in **A**). At P2, NFIX expression was detected in the wild-type dentate gyrus (arrow in **C**), CA3 (double arrowhead in **C**) and CA1 pyramidal cell layers (arrowhead in **C**) but was detected only in the dentate gyrus at P21 (arrow in **E**). In adult wild-type mice (**G**), NFIX was most strongly expressed by cells in the SGZ of the dentate gyrus (arrowheads in **G’**). The same expression pattern was observed in *Nfix* ^+/−^ mice (**B, D, F, H, H’**), although the level of NFIX expression was much lower than in wild-type mice at all ages. The specificity of the NFIX antibody is demonstrated by the absence of immunoreactivity in *Nfix^−/−^* knockout tissue at E17 (**J,** compare to panel **I**). **G’** and **H’** are magnified views of the boxed regions in **G** and **H** respectively. Scale bar (in **J**): **A, B, I, J** 150 µm; **C, D** 250 µm; **E, F, G, H** 500 µm; **G’, H’** 50 µm.

We first assessed NFIX expression in both wild-type and heterozygous mice at embryonic, postnatal and adult ages. At E14 in wild-type mice, NFIX was expressed in two regions of the hippocampal ventricular zone, the ammonic neuroepithelium and the dentate neuroepithelium, as well as by post-mitotic neurons in the developing hippocampal anlage (Fig. 1*A*). At E14 in *Nfix* ^+/−^ mice, NFIX was also detected in these regions but at a reduced level (Fig. 1*B*). At P2 in wild-type mice, NFIX expression was detected in the dentate gyrus and the CA3 and CA1 regions of the hippocampus, but by P21 its expression was largely restricted to the dentate gyrus (Fig. 1*C, E*), consistent with previous reports [13]. This same expression pattern was also observed in *Nfix* ^+/−^ mice at P2 and P21, albeit at a lower level (Fig. 1*D, F*). In adult wild-type mice, NFIX was relatively weakly expressed within the granule cell layer of the dentate gyrus, but was strongly expressed by cells in the SGZ. These cells are likely to be the neural progenitor cells of the dentate gyrus (Fig. 1*G, G* *’*). In adult *Nfix* ^+/−^ mice, expression of NFIX was markedly reduced in both the granule cell layer and the SGZ when compared to the pattern in wild-type mice (Fig. 1*H, H* *’*). The specificity of the NFIX antibody used in these experiments was shown by the absence of immunoreactivity in *Nfix* knockout tissue at E17 (Fig. 1*J*). These data demonstrate that *Nfix* ^+/−^ mice have reduced expression of NFIX within the hippocampus both during development and in adulthood.

### Embryonic Development of the Hippocampus is Delayed in *Nfix^+/−^* Mice

**Figure 2:**
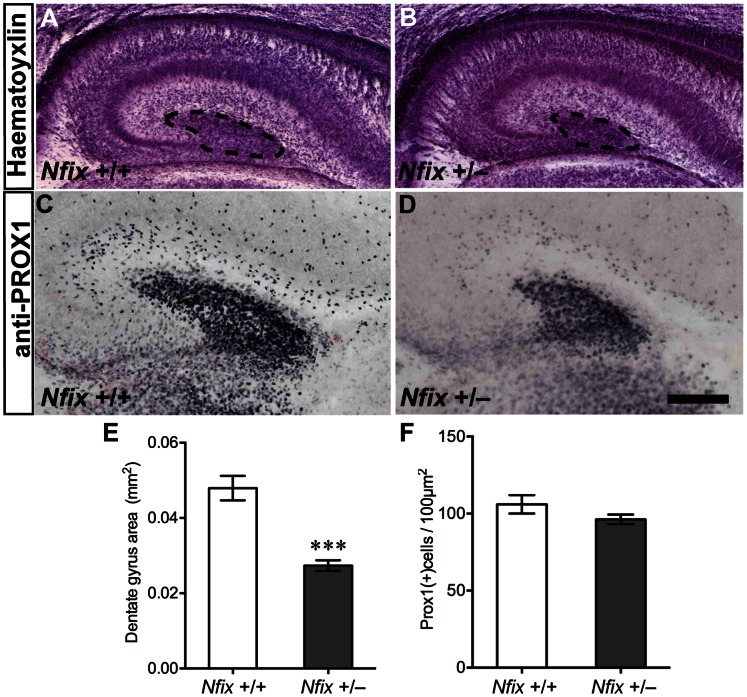
The dentate gyrus is reduced in E18 *Nfix* ^+/−^ mice. Haematoxylin (**A, B)** and anti-PROX1 staining (**C, D**) of E18 wild-type and *Nfix* ^+/−^ coronal brain sections. The overall morphology of the hippocampus in *Nfix* ^+/−^ mice at E18 (**B**) was normal compared with that of wild-type mice (**A**), except that the dentate gyrus appeared smaller (dentate gyrus delineated by dashed lines in **A, B**). PROX1 immunohistochemistry (**C**, **D**) confirmed that the overall dentate gyrus area was smaller in *Nfix* ^+/−^ mice at this age (***p<0.001, **E**). No difference was detected for the number of PROX1-positive cells per unit area of the dentate gyrus (*p = *0.19, **F**). Scale bar (in **D**): **A, B** 200 µm; **C, D** 100 µm.

Given that *Nfix* ^+/−^ mice had reduced hippocampal expression of NFIX we hypothesised that the embryonic development of the hippocampus in these mice would be delayed. To test this hypothesis we first performed haematoxylin staining on *Nfix^+/−^* and wild-type tissue at E18. At this age, although the CA pyramidal cell layers in heterozygous mice were grossly normal, the emerging dentate gyrus appeared smaller than in the wild-type controls (Fig. 2*A,B*). To quantify the size of the dentate gyrus in *Nfix^+/−^* mice we performed immunohistochemistry for PROX1, a marker for dentate granule neurons within the hippocampus. Immunostaining for PROX1 delineated the emerging dentate gyrus in both wild-type and *Nfix^+/−^* mice at this age (Fig. 2*C,D*; [26]). In *Nfix^+/−^* mice the dentate gyrus did not extend as far laterally as in wild-type mice, and quantification of the area encompassed by PROX1-positive cells confirmed that overall dentate gyrus size was reduced in these animals (*t*_8_ = 5.81; *p*<0.001; Fig. 2*E*), although no difference in the number of PROX1-positive cells per unit area of the dentate gyrus was detected (*t*_8_ = 1.45; *p = *0.19; Fig. 2*F*).

**Figure 3:**
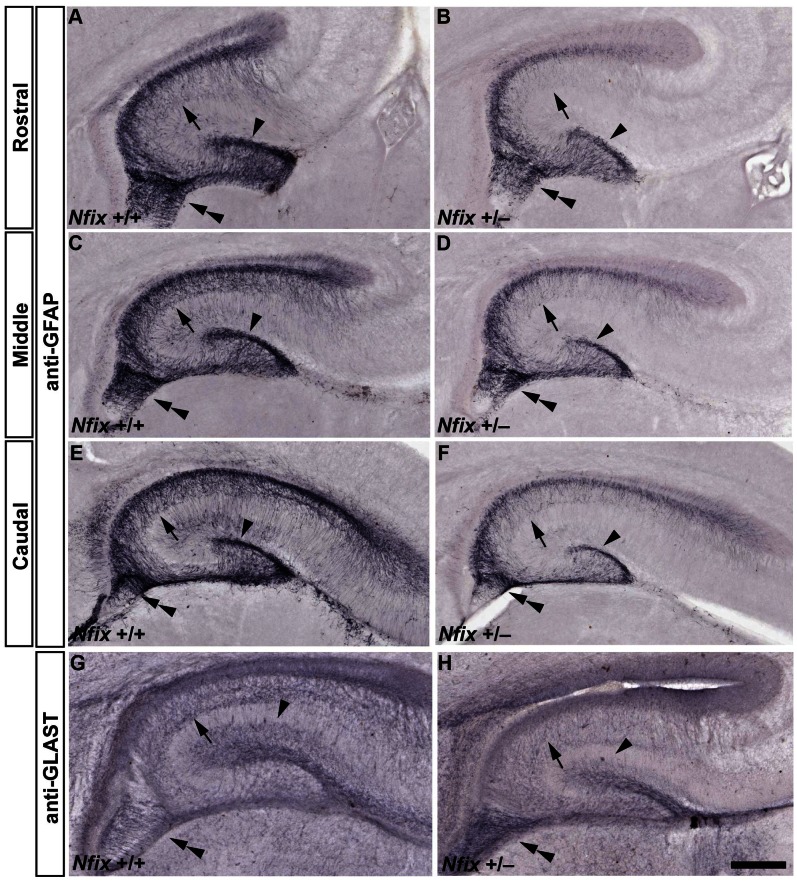
GFAP and GLAST immunoreactivity is reduced in E18 *Nfix* ^+/−^ mice. Anti-GFAP (**A–F)** and anti-GLAST staining (**G**, **H**) of E18 wild-type and *Nfix* ^+/−^ coronal brain sections. There was reduced GFAP immunoreactivity in the hippocampus of *Nfix* ^+/−^ mice (**B, D, F)** compared to that of their wild-type littermates at E18 (**A, C, E**). This reduction in GFAP staining was evident along the rostrocaudal axis of the hippocampus in the supragranular glial bundle (arrowhead), fimbrial glial bundle (double arrowhead) and CA regions (arrow). There was also reduced staining for GLAST in the CA regions and supragranular glial bundle of *Nfix* ^+/−^ mice at E18 (compare panels **G** and **H)**, although immunoreactivity appeared darker in the fimbrial glial bundle. Scale bar (in **H**): **A–H** 250 µm.

We next assessed the expression of glial markers in the hippocampus of E18 *Nfix^+/−^* mice. Glial cells are critical for the normal morphogenesis of the hippocampus and are derived from progenitor cells within two regions of the hippocampal ventricular zone from E15, the ammonic neuroepithelium and the fimbrioglial epithelium [10], [27]. The expression of the astroglial marker GFAP is widely used to analyse glial differentiation within the hippocampal anlage [28]. Based on GFAP immunohistochemistry at E18 we found that the supragranular glial bundle, arising from the ammonic neuroepithelium and the fimbrial glia bundle, arising from the fimbrioglial epithelium, had formed in the hippocampus of *Nfix* ^+/−^ mice at this age (Fig. 3). However, the intensity of GFAP staining was reduced in these areas compared to wild-type mice, in particular the number of GFAP-positive fibres localising to the CA regions in the hippocampus in heterozygous mice was reduced (Fig. 3*B, D, F*). This reduction in GFAP expression in *Nfix* ^+/−^ mice was observed along the rostral-caudal axis of the hippocampus in all of the brains that were examined and is consistent with previous reports showing that NFIX is able to bind to the promoter region of the *Gfap* gene to activate its expression [16]. Furthermore, we also analysed the expression of the astroglial marker GLAST at E18. The expression of GLAST was also reduced in the supragranular glial bundle and CA regions of *Nfix* ^+/−^ mice compared to wild-type controls, although not in the fimbrial glial bundle (Fig. 3*G, H*). Taken together, the reduced size of the dentate gyrus and the reduced expression of glial markers indicate that both neuronal and glial development were delayed in E18 *Nfix* ^+/−^ mice.

### Hippocampal Morphology of *Nfix^+/−^* Mice Remains Abnormal during the Early Postnatal Period

**Figure 4:**
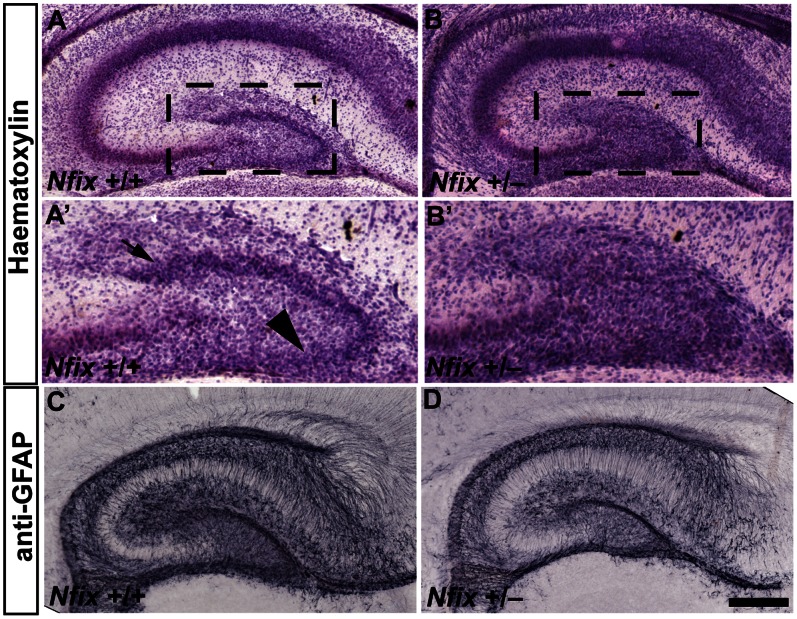
Hippocampal development is delayed in P2 *Nfix* ^+/−^ mice. Haematoxylin (**A**–**B’**) and anti-GFAP staining (**C** and **D**) of P2 wild-type and *Nfix* ^+/−^ coronal brain sections. In wild-type mice (**A** and **A’**) the suprapyramidal blade (arrow in **A’**) and infrapyramidal blade (arrowhead in **A’**) of the dentate gyrus had begun to resolve, whereas in *Nfix* ^+/−^ mice (**B** and **B’**) the dentate gyrus remained amorphous. GFAP staining was also reduced in *Nfix* ^+/−^ mice (**D**) relative to wild-type controls at P2 (**C**). **A’** and **B’** are magnified views of the boxed regions in **A** and **B** respectively. Scale bar (in **D**): **A**, **B, C, D** 250 µm; **A’**, **B’** 50 µm.

**Figure 5:**
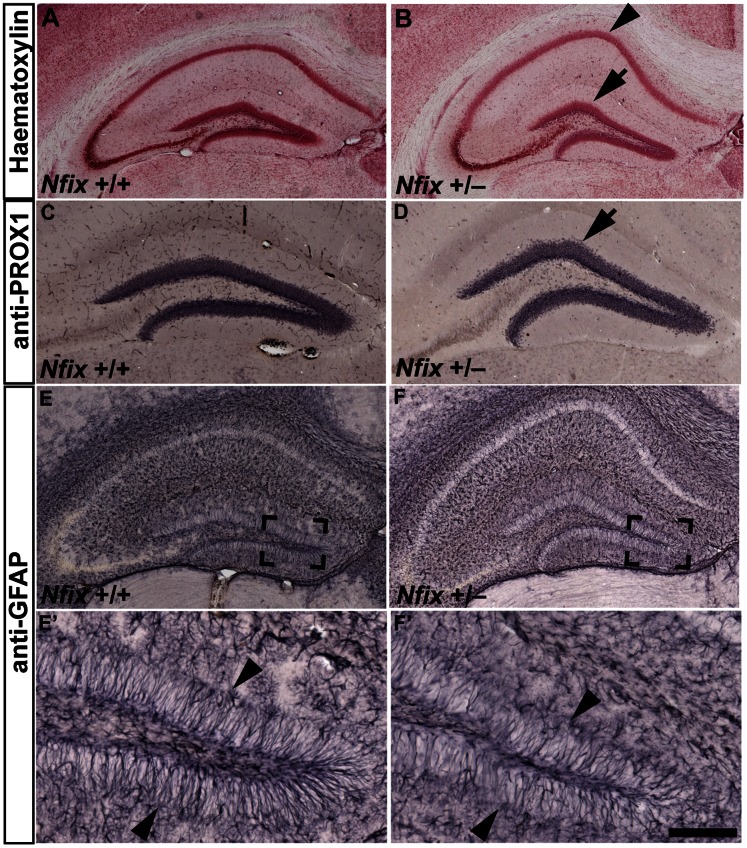
Hippocampal morphology is aberrant in P21 *Nfix* ^+/−^ mice. Haematoxylin (**A** and **B)**, anti-PROX1 (**C** and **D**) and anti-GFAP staining (**E**–**F’**) of coronal sections of P21 wild-type and *Nfix* ^+/−^ brains. In *Nfix* ^+/−^ mice the CA1 pyramidal cell layer had a more pronounced dorsal curvature (arrowhead in **B**) than in wild-type mice (**A**). The dentate gyrus was also misshapen in that the lateral region of the suprapyramidal blade met the medial region at a more acute angle (arrow in **B** and **D)** than in wild-type mice (**A** and **C**). Although no gross difference in GFAP staining was apparent at P21 (**E** and **F**), there appeared fewer GFAP-positive radial fibres in the dentate gyrus of *Nfix* ^+/−^ mice (arrowheads in **F’**) than wild-type mice **(**arrowheads in **E’**). **E’** and **F’** are magnified views of the boxed regions in **E** and **F** respectively. Scale bar (in **F’**): **A, B** 275 µm; **C**, **D** 180 µm; **E, F** 250 µm; **E’, F’** 50 µm.

We next assessed the morphology of the hippocampus in *Nfix* ^+/−^ mice at P2. Haematoxylin staining revealed that the dentate gyrus of wild-type mice had resolved into distinct suprapyramidal and infrapyramidal blades at this age, whereas in *Nfix* ^+/−^ mice the dentate gyrus remained amorphous (Fig. 4*A–B* *’*). Furthermore, GFAP expression was reduced in *Nfix* ^+/−^ mice relative to wild-type mice at P2, demonstrating that hippocampal development remains disrupted in *Nfix* ^+/−^ mice shortly after birth (Fig. 4*C, D*). At P21, haematoxylin staining revealed that the dorsal curvature of the CA1 pyramidal cell layer was more pronounced in *Nfix* ^+/−^ mice than in wild-type animals (Fig. 5*B*). This abnormal dorsal curvature of the CA1 layer has previously been observed in homozygous *Nfix* knockout mice [11], [13]. Immunostaining for PROX1 also revealed that the dentate gyrus was misshapen in *Nfix* ^+/−^ mice at P21, in that the lateral region of the suprapyramidal blade met the medial region of the suprapyramidal blade at a more acute angle than in wild-type mice (arrow in Fig. 5*D*). Furthermore, although GFAP expression was grossly normal in *Nfix* ^+/−^ mice by P21, radial GFAP-positive processes extending from type 1 progenitor cells within the SGZ of the dentate gyrus appeared fewer in number than in wild-type mice at this age (Fig. 5*E, F*). Collectively these data demonstrate that reduced expression of NFIX during development culminates in abnormal hippocampal morphology during the early postnatal period.

### The Hippocampus of Adult *Nfix^+/−^* Mice Exhibits Subtle Morphological Abnormalities

**Figure 6:**
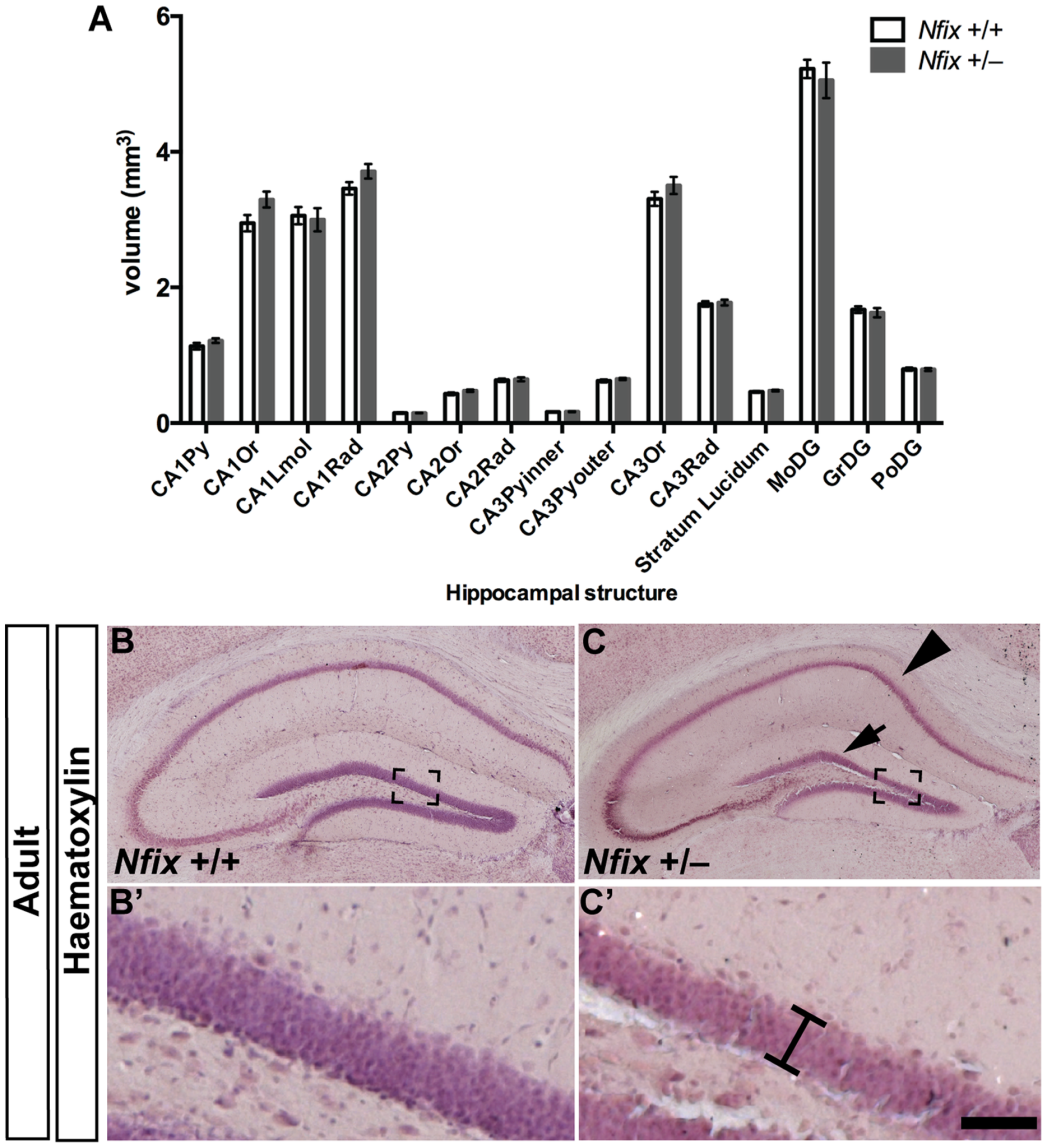
The hippocampus of adult *Nfix* ^+/−^ mice remains misshapen. No gross differences in the volume of hippocampal structures were detected between adult wild-type and *Nfix* ^+/−^ mice (**A**) using magnetic resonance imaging at a resolution of 30 µm (*p*>0.5). Haematoxylin staining of coronal brain sections (**B** and **C**) revealed that the CA1 pyramidal cell layer (arrowhead in **C**) and dentate gyrus (arrow in **C**) remained misshapen in adult *Nfix^+/−^* mice. Moreover the suprapyramidal blade of the dentate gyrus appeared thinner in adult *Nfix* ^+/−^ mice (**C’**) compared with wild-type mice (**B’**). **B’** and **C’** are magnified views of the boxed regions in **B** and **C** respectively. Scale bar (in **C’**): **B**, **C** 500 µm; **B’**, **C’** 70 µm. Abbreviations: CA = Cornu ammonis; Py = pyramidal layer; Or = oriens layer; Rad = radiatum layer; MoDG = molecular layer of the dentate gyrus; GrDG = granule layer of the dentate gyrus; PoDG = polymorph layer of the dentate gyrus.

**Figure 7:**
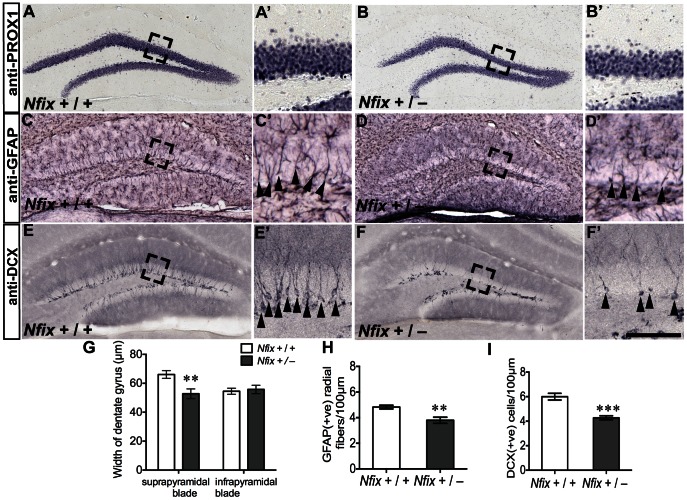
Hippocampal neurogenesis is impaired in adult *Nfix* ^+/−^ mice. Anti-PROX1 (**A**–**B’**), anti-GFAP (**C**–**D’**) and anti-DCX (**E**–**F’**) staining of coronal sections of adult wild-type and *Nfix* ^+/−^ brains. Immunostaining for PROX1 and subsequent quantification (**A**–**B’**) revealed that the thickness of the suprapyramidal blade of the dentate gyrus was reduced in adult *Nfix* ^+/−^ mice compared with wild-type mice (**p<0.01; **G**). There were also fewer GFAP-positive radial fibres (**p<0.01; **H**) and DCX-positive neurons (***p<0.001*;* **I**) per unit length of the dentate gyrus in adult *Nfix* ^+/−^ mice. **A’, B’, C’, D’, E’** and **F’** are magnified views of the boxed regions in **A, B, C, D, E** and **F** respectively. Scale bar (in **F’**) **A, B, C, D, E, F** 300 µm; **A’, B’, C’, D’, E’, F’** 75 µm.

Unlike the full *Nfix* knockout mouse that dies at P21, *Nfix* ^+/−^ mice survive to adulthood [13], [29], making them an ideal model to investigate whether reduced levels of NFIX expression developmentally and within the adult cause abnormal hippocampal morphology and function. We first addressed the issue of hippocampal morphology within adult *Nfix^+/−^* mice by performing magnetic resonance imaging, at a resolution of 30 µm, to determine if there were any gross volumetric differences in hippocampal structures between adult wild-type and *Nfix^+/−^* mice. Analysis of these scans did not detect any effect of genotype on the volume of hippocampal structures (*F*_1, 20_ = 0.46; *p*>0.5; Fig. 6*A*). However, these data do not preclude subtle abnormalities within the hippocampal morphology of *Nfix^+/−^* mice that could not be determined at this resolution. Moreover, the analysis of these scans was constrained to determine volumetric differences and, as such, structural changes that do not give rise to such differences may not be apparent within this paradigm. We therefore also processed adult wild-type and adult *Nfix* ^+/−^ brains for histological and immunohistochemical analysis. Haematoxylin staining revealed that, similar to the phenotype at P21, the CA1 pyramidal cell layer had a more pronounced dorsal curvature in adult *Nfix* ^+/−^ mice than in wild-type controls (Fig. 6*B, C*). The dentate gyrus also remained misshapen in adult *Nfix* ^+/−^ mice; however, unlike at P21, immunostaining for PROX1 revealed that the suprapyramidal blade of the dentate gyrus was thinner in adult *Nfix* ^+/−^ mice than in wild-type controls (*t_22_* = 3.26; *p<*0.01; Fig. 7A, B, *G*). Taken together, these data demonstrate that the reduced expression of NFIX throughout development affects the morphology of the hippocampus in the adult brain.

### Neurogenesis is Disrupted in the Hippocampus of Adult *Nfix^+/−^* Mice

The hippocampus is critical for learning and memory, processes which are regulated, in part, by the birth of new neurons from progenitors within the SGZ of the hippocampal dentate gyrus during adulthood [30]. Type 1 radial progenitor cells within the SGZ can be identified as they extend a GFAP-positive radial process into the granule cell layer of the dentate gyrus [31]. Earlier we had shown that cells in the SGZ express NFIX (Fig. 1*G* *’*,*H’*), and that at P21 there were fewer radially oriented GFAP-positive fibres in the dentate gyrus of *Nfix* ^+/−^ mice than in wild-type controls (Fig. 5*E* *’*,*F’*). These observations, coupled with our data demonstrating that the suprapyramidal blade of the dentate gyrus was thinner in adult *Nfix* ^+/−^ mice (Fig. 7*G*), led us to postulate that the production of new dentate granule neurons might also be disrupted in adult *Nfix* ^+/−^ mice.

To address this possibility we first examined the expression of GFAP. In adult wild-type mice, radially oriented GFAP-positive processes of progenitor cells extended from the SGZ across the breadth of the granule cell layer (Fig. 7*C, C* *’*). In adult *Nfix* ^+/−^ mice (Fig. 7*D, D* *’*), cell counts revealed that there were fewer GFAP-positive radial fibres per unit length of the dentate gyrus in *Nfix* ^+/−^ mice than in wild-type controls (*t*_11_ = 3.54; *p*<0.005; Fig. 7*H*). SGZ progenitor cells give rise to immature neurons that express the microtubule-associated protein DCX [32]. In wild-type mice, DCX-positive immature neurons lined the length of the dentate gyrus and extended a dense array of dendrites into the molecular layer of the hippocampus (Fig. 7*E, E* *’*). However, in adult *Nfix* ^+/−^ mice there were significantly fewer DCX-positive cells than in wild-type mice (*t*_11_ = 5.59; *p<*0.001; Fig. 7*F, F* *’, I*). Collectively, these findings reveal that, in addition to structural abnormalities of the hippocampus, mice heterozygous for the *Nfix* gene possess reduced numbers of radial type 1 progenitor cells, and display impairments in the production of new dentate granule neurons within the adult brain.

### Adult *Nfix^+/−^* Mice Exhibit Normal Behaviour on Tests of Motor Function and Anxiety

**Figure 8:**
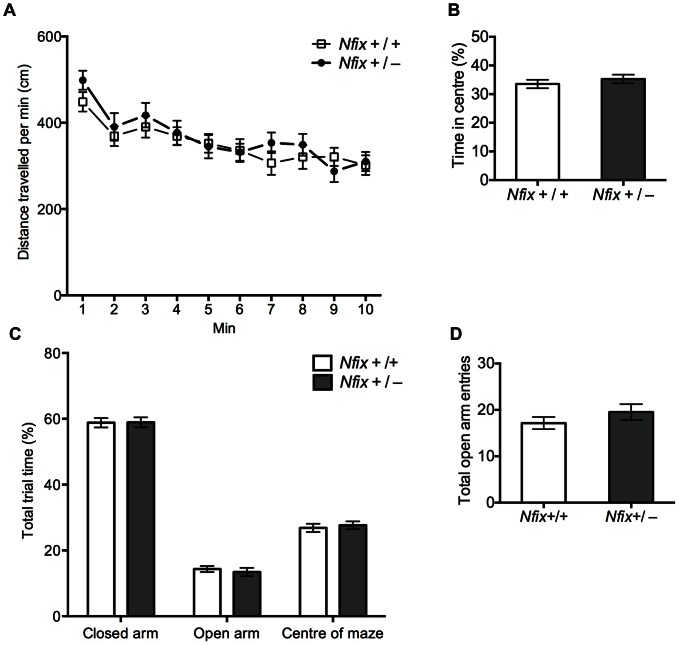
*Nfix^+/−^* mice exhibit normal motor function and anxiety-related behaviour. Performance of adult wild-type and *Nfix* ^+/−^ mice during the open-field test (**A, B**) and the elevated plus maze (**C, D**). In the open-field test there was no effect of genotype on the distance travelled over the 10 minute test period (*p*>0.5; **A**), or on time spent in the centre of the test apparatus (*p* = 0.43; **B**). In the elevated plus maze no effect of genotype was observed on the percentage of total trial time spent on the open arms, closed arms or the centre position of the maze (*p*>0.5; **C**), or on number of open arm entries (*p* = 0.29; **D**). (*Nfix* ^+/+^: n = 20, *Nfix* ^+/−^: n = 26).

Hippocampal lesions and impaired hippocampal neurogenesis result in reduced spatial learning and memory performance in rodents and in humans [33]–[35]. The aberrant hippocampal morphology of adult *Nfix* ^+/−^ mice, and the fact that adult neurogenesis was reduced in these mice, suggested that they might display reduced performance on hippocampal-specific spatial learning and memory tasks. To assess this we performed a battery of tests to determine whether the mice were phenotypically normal in other behavioural domains. First, we conducted a general primary observation screen (SHIRPA; modified from [23]). No differences were detected between *Nfix* ^+/−^ mice and wild-type mice during this screen, demonstrating that the gross morphology and behaviour of the *Nfix* ^+/−^ mice was normal (Tables 1–3). We then undertook an open-field test, which is used to measure aspects of behaviour when a mouse is placed in a novel environment from which it cannot escape. In this test, the exploratory behaviour and motor function of the *Nfix* ^+/−^ mice was normal (Fig. 8A). Finally, we used the elevated plus maze to assess anxiety-related behaviour, revealing there to be no effect of genotype on time spent in the open arm or any other position in the maze (*F*_2, 44_ = 0.00, *p*>0.5; Fig. 8*C*), or on the number of open-arm entries (*t*_44_ = 1.06, *p* = 0.29; Fig. 8*D*). This indicated that *Nfix^+/−^* mice have similar anxiety levels to those of wild-type mice. This conclusion was also supported by data from the open-field test where *Nfix^+/−^* mice spent as much time as wild-type mice in the centre of the test apparatus, which is highly aversive for rodents due to the increased light intensity (*t*_42_ = 0.80, *p* = 0.43; Fig. 8*B*). Collectively, the data from the SHIRPA screen, open-field test and elevated plus maze indicated that *Nfix^+/−^* mice display normal gross morphology and behaviour, motor function and anxiety-related behaviour, enabling us to investigate spatial learning and memory within our chosen behavioural task (the Morris water maze) without these potential confounds.

**Table 1:** Behaviour and appearance in viewing jar

Behaviour/Physical Trait	Criteria	*Nfix* ^+/+^	*Nfix* ^+/−^
Body position	Inactive	10%	16%
	Active	60%	44%
	Very Active	30%	40%
Tremor	Present	0%	0%
Palpebral closure	Present	100%	100%
Coat appearance	Normal	100%	100%
Whiskers	Present	96%	100%
Lacrimation	Present	100%	100%
Defecation	Present	40%	56%

Wild-type mice and *Nfix* ^+/−^ mice were placed in a large Perspex viewing jar (15 cm diameter) for approximately 2 minutes. Aspects of physical appearance and behaviour were scored during this time. No large differences were apparent between genotypes (*Nfix* ^+/+^, n = 20; *Nfix* ^+/−^ n = 26).

**Table 2:** Behaviour above viewing jar.

Behaviour/Physical Trait	Criteria	*Nfix* ^+/+^	*Nfix* ^+/−^
Positional passivity	Struggled when held by tail	100%	100%
Trunk curl	Present	100%	100%
Visual placing	Present	100%	100%
Righting reflex	Present	100%	100%
Pinnal reflex	Present	85%	96%
Vocalisation	Present	50%	60%
Morphology	Normal	100%	100%
Tail pinch	Response	100%	100%
Biting	Present	5%	4%
Limb grasping	Present	65%	54%

Wild-type and *Nfix* ^+/−^ mice were held above the viewing jar by the base of the tail whilst a number of physical, behavioural and reflexes were scored. No large differences were apparent between genotypes (*Nfix* ^+/+^, n = 20; *Nfix* ^+/−^, n = 26).

**Table 3:** Body weight.

Behaviour/Physical Trait	Descriptive Term	*Nfix* ^+/+^	*Nfix* ^+/−^
Weight	Female (g)	21.37±0.782	21.46±0.782
	Male (g)	26.28±0.871	28.71±0.734

The weights (g) of wild-type mice and *Nfix* ^+/−^ mice were recorded. There was weak evidence (*p* = 0.08) to suggest that male *Nfix^+^* ^/−^ mice were on average heavier than male wild-type mice. There was no evidence that female *Nfix* ^+/−^ mice were heavier than female wild-type mice. The age that mice were weighed at varied from P63–P73. To account for this source of variation, ‘age-at-weigh-in’ was added as a covariate during the statistical analysis of these data (*Nfix* ^+/+^, n = 20, 9 male, 11 female; *Nfix* ^+/−^, n = 26, 12 male, 14 female).

### Adult *Nfix^+/−^* Mice have Impaired Spatial Learning and Memory

**Figure 9:**
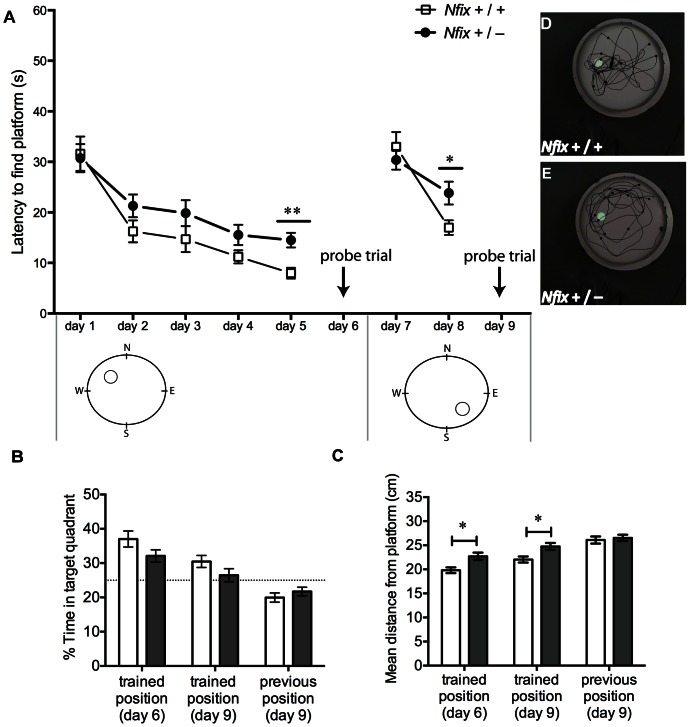
*Nfix^+/−^* mice have impaired spatial learning and memory. Training latencies (**A**) and probe trial performances (**B–E**) of adult wild-type and *Nfix* ^+/−^ mice during the Morris water maze task. Over the initial 5 days of hidden-platform training (days 1–5) *Nfix* ^+/−^ mice took significantly longer, on average, than wild-type mice to find the platform (**p<0.01), taking almost twice as long on training day 5 (**p<0.01; **A**). During the reversal learning procedure (days 7–8) *Nfix* ^+/−^ mice took longer to find the platform on training day 8 (*p<0.05; **A**). The average search path of *Nfix* ^+/−^ mice was further away from the trained position of platform during the probe trial on day 6 and day 9 (*p<0.05; **C**). A similar trend was observed for time spent in target quadrant (**B**). A representative performance of a wild-type mouse and a *Nfix* ^+/−^ mouse during the probe trial on day 6 is pictured in (**D**) and (**E**) respectively. (*Nfix* ^+/+^: n = 20, *Nfix* ^+/−^: n = 26).

The Morris water maze is the most frequently used method for assessing spatial learning and memory in rodents [36]. In this test, rodents must use visual cues outside the maze to triangulate the position of a submerged platform. We found that over the initial 5 days of hidden-platform training *Nfix* ^+/−^ mice took significantly longer, on average, to locate the submerged platform than wild-type mice (*F*_1, 176_ = 7.32, *p*<0.01). Specifically, on day 5 of testing *Nfix* ^+/−^ mice took approximately twice as long to find the platform as wild-type mice (*t*_44_ = 3.45, *p*<0.01; Fig. 9*A*). Subsequently, during the day 6 probe trial, while no effect of genotype was found for time spent in the target quadrant (*t*_132_ = 1.96, *p = *0.16; Fig. 9*B*), the average search path of the *Nfix* ^+/−^ mice during the 1 minute probe trial was further from the trained platform position than the search path of wild-type mice (*t*_132_ = 2.87, *p*<0.05; Fig. 9*C*). Furthermore, during reversal learning on days 7–8, *Nfix* ^+/−^ mice took longer to find the platform on day 8 of testing (*t*_44_ = 2.38; *p*<0.05; Fig. 9*A*). The increased training latency of *Nfix* ^+/−^ mice on day 8 was reflected in impaired probe trial performance on day 9 (*t*_132_ = 2.72, *p*<0.05; Fig. 9*C*), with *Nfix* ^+/−^ mice swimming further from the trained platform position than wild-type mice, although no significant effect was detected for time spent in the target quadrant (*t*_132_ = 1.61; *p = *0.33; Fig. 9*B*).

To determine if any other factors, including gender, inter-cohort effects and swimming ability, could have influenced the results from the Morris water maze test, we performed further statistical analyses of the data from the first probe trial on day 6. We found that there was no cohort x genotype interaction, indicating that the reduced performance of the *Nfix* ^+/−^ mice was consistent across the three cohorts of mice that were tested during this project (*F*_2, 40_ = 1.47, *p = *0.24). We also found no effect of genotype on average swim velocity during the test, demonstrating that the increased training latencies of the *Nfix^+/−^* mice was due to a spatial learning defect and was not related to the swimming ability of these mice (*t*_44_ = 0.41, *p*>0.5; *Nfix* ^+/+^19.74±0.4077 cm/s and *Nfix* ^+/−^19.47±0.4679 cm/s). Together these data demonstrate that *Nfix* ^+/−^ mice have a specific deficit in spatial learning and memory.

## Discussion

In this report we present three major findings. Firstly, we demonstrate that the embryonic and early postnatal development of the hippocampus is disrupted in *Nfix* ^+/−^ mice. Secondly we reveal that the hippocampal dentate gyrus of adult *Nfix* ^+/−^ mice has reduced levels of neurogenesis, and that the CA1 region is misshapen. Lastly, we demonstrate that adult *Nfix* ^+/−^ mice display impaired performance on a hippocampal-dependent spatial learning and memory task. These data reveal that normal expression of *Nfix* during development is essential for the formation and function of the hippocampus in the mature nervous system.

This is the first study to show that reducing the expression of NFIX during development by heterozygosity for the *Nfix* gene affects the morphology and function of the hippocampus in the adult mouse brain. Previous studies examining the homozygous *Nfix* knockout mouse have shown that NFIX is important for embryonic and early postnatal neurogenesis and gliogenesis within the hippocampus [11], [13]. However, as homozygous *Nfix* knockout mice die at P21, it was unclear whether disrupting NFIX expression during development would affect the hippocampal-dependent behaviour of adult mice. This is especially relevant given the extensive recovery in neuronal and glial differentiation within the homozygous *Nfix* knockout dentate gyrus by P10 [11]. Our data demonstrate that reduced NFIX expression during development results in subtle morphological abnormalities in the dentate gyrus and CA regions of adult mice, and that this is potentially correlated with reduced performance in a hippocampal-dependent spatial learning and memory task, the Morris water maze.

The findings from this study have also improved our understanding of how NFIX regulates the early development of the hippocampus. Recent work examining the phenotype of the *Nfix* knockout mouse has demonstrated that NFIX regulates the early development of the hippocampus by promoting the differentiation of progenitor cells within the hippocampal ventricular zone, in part via the repression of the transcription factor SOX9 that maintains progenitor cell identity [11]. In the absence of NFIX, ventricular zone progenitor cells are retained in a progenitor state for longer than in wild-type mice, leading to a delay in the production of hippocampal CA neurons and dentate granule neurons, as well as hippocampal glia [11], [13]. Our study has furthered our understanding of how NFIX regulates hippocampal development by demonstrating that gene dosage of NFIX is also an important determinant of progenitor cell differentiation. Specifically, reduced expression of NFIX in *Nfix* ^+/−^ mice led to a delay in glial differentiation within the developing hippocampus. Furthermore, the reduced size of the hippocampal dentate gyrus in *Nfix* ^+/−^ mice at E18 indicates that the production of dentate granule neurons is also delayed in these animals. Therefore gene dosage of NFIX and progenitor cell differentiation within the hippocampus are inextricably linked.

Our results have also indicated a role for NFIX in regulating adult neurogenesis within the dentate gyrus. Previously it has been shown that NFIX, as well as NFIA and NFIB, are expressed within the SGZ neurogenic niche of the dentate gyrus in adult mice [13], [14]. However, the question of whether *Nfi* genes have a role in regulating adult neurogenesis has so far remained unexplored. Here we have demonstrated that the production of new neurons within the hippocampal dentate gyrus of adult *Nfix* ^+/−^ mice is impaired, with fewer radial type 1 SGZ progenitor cells and DCX-positive immature neurons in the dentate gyrus of these mice than in wild-type animals, suggestive of a potential role for NFIX in regulating adult neurogenesis within the hippocampus. Interestingly, as most studies examining the behavioural effects of ablating adult hippocampal neurogenesis have implicated the process in spatial learning and memory [30], [37] the impairment in neurogenesis in adult *Nfix* ^+/−^ mice may have contributed, together with the structural abnormalities of the hippocampus, to the reduced performance of these mice during the Morris water maze task.

One point to consider in light of these data is that, while we have demonstrated that *Nfix^+/−^* mice had reduced expression of NFIX within the developing hippocampus, this reduction was evident in other areas of the central nervous system, as this line affects global *Nfix* expression, rather than being hippocampal-specific (Fig. S1). This is an important caveat to consider because NFIX, as alluded to earlier, has previously been shown to be important for the development of other regions of the nervous system, including the formation of the corpus callosum, subventricular zone (SVZ) and cerebellum [6], [13], [29]. Thus, there is the possibility that the impaired performance of *Nfix* ^+/−^ mice in the Morris water maze test could have been due to the aberrant formation of these structures, rather than the hippocampus. However, we believe this to be unlikely for a number of reasons. Firstly, the corpus callosum and SVZ are not thought to be central to performance in the Morris water maze [38]. Moreover, although the cerebellum has been shown to be important for procedural aspects of the Morris water maze task, such as search strategy and motor coordination [39], [40], the search strategy of *Nfix* ^+/−^ mice was normal, as was the motor coordination of these mice when this was examined during the open-field test. Finally, if *Nfix* ^+/−^ mice had any other neuroanatomical defects these would have culminated in aberrant behaviour that should have been detected in the SHIRPA screen. Therefore, although we cannot draw any definitive causal links between the hippocampal phenotype of *Nfix* ^+/−^ mice and their behavioural phenotype within this paradigm, our data are strongly supportive of the notion that the reduced performance of *Nfix* ^+/−^ mice in the Morris water maze is due to the impaired hippocampal function of these mice.

Although our data clearly indicate that *Nfix* ^+/−^ mice exhibit a deficit in hippocampal-specific learning and memory, it is also difficult to determine if this phenotype arose solely from aberrant hippocampal morphogenesis during development or was also due to distinct roles of NFIX in the adult brain. Previously, we have shown that the formation of the SGZ is impaired in homozygous *Nfix* knockout mice. In these mice at P15 there are far fewer radially oriented GFAP-positive and DCX-positive fibres, as well as a disorganisation of SOX2-positive progenitors within the hilar region of the hippocampus [11], suggesting that NFIX plays an important role in the development of this neurogenic niche during the early postnatal period. We demonstrate here that *Nfix* ^+/−^ mice have a phenotype similar to this at P21. However, as cells within the SGZ also strongly express NFIX, it is likely that NFIX has a cell-autonomous role in regulating hippocampal neurogenesis in the adult brain. In the future, the use of a conditional *Nfix* allele, coupled with the use of an inducible cre-recombinase under the control of neural progenitor cell-specific promoter, such as the nestin-cre ERT2 line [41], will enable the ablation of *Nfix* specifically from progenitor cells within the adult brain. This will enable us to parse the role of NFIX in development and in the adult, allowing more precise determination of the role played by this transcription factor in adult dentate gyrus neurogenesis.

Finally, the results from this study may also provide insights into human congenital disorders caused by *Nfi* deletions. Recently, it was shown that haploinsufficiency for *NFIX* is one of the causative factors for both Sotos syndrome and Marshall-Smith syndromes [18]–[20]. Both of these conditions are characterised by overgrowth, musculoskeletal abnormalities, abnormal behaviour including autistic traits, and mild to moderate learning difficulties. Magnetic resonance imaging analyses have shown that these individuals recapitulate some of the gross defects observed in the homozygous *Nfix* knockout mouse, including hypoplasia of the corpus callosum and ventricular dilation [42], [43]. Although no gross defects in hippocampal morphology were reported in these studies, our results suggest that individuals with these disorders may exhibit subtle defects in hippocampal morphology and hippocampal-dependent behaviour, and that this could contribute to their neurological deficits.

## Supporting Information

Figure S1 ***Nfix*** **^+/−^ mice exhibit a reduction of NFIX expression in the cerebellum and lateral ventricles.** Anti-NFIX staining of wild-type and *Nfix* ^+/−^ brain sections at P5 (**A–F**). NFIX expression in heterozygote animals appeared relatively normal within the neocortex at this age (**A, B**), but NFIX expression was reduced within the periventricular zone of the lateral ventricles (**C, D**) and within the cerebellum (**E, F**). Scale bar (in **F**): **A–D** 50 µm; **E, F** 100 µm.
(TIF)
