# Supplementary figures and images for: Heterozygosity for Nuclear Factor One X Affects Hippocampal-Dependent Behaviour in Mice
Source: PLoS One. 2013 Jun 11;8(6):e65478. doi: 10.1371/journal.pone.0065478 (PMC3679126; doi:10.1371/journal.pone.0065478)

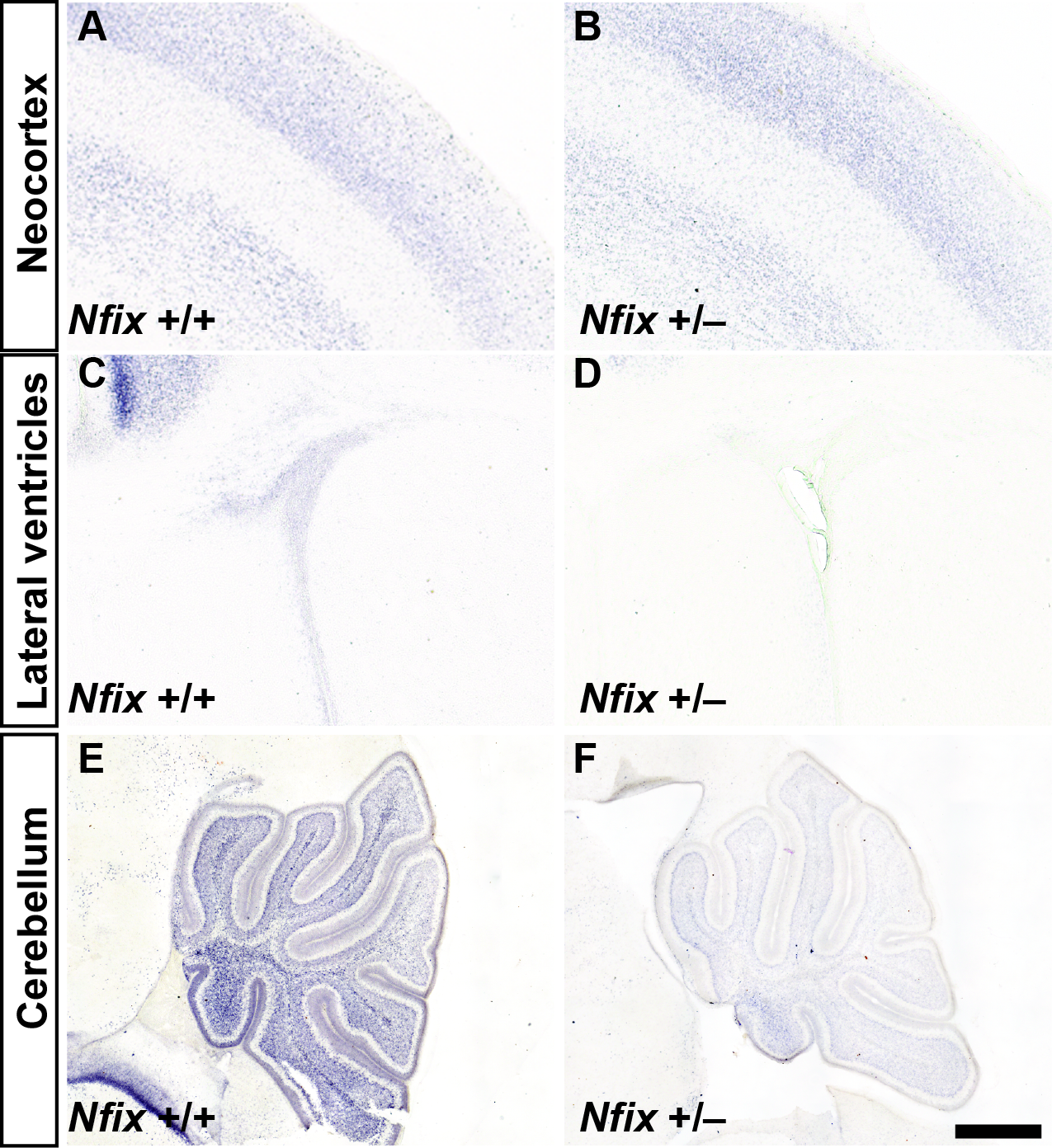

Supplement: Figure S1 — Nfix +/− mice exhibit a reduction of NFIX expression in the cerebellum and lateral ventricles. Anti-NFIX staining of wild-type and Nfix +/− brain sections at P5 (A–F). NFIX expression in heterozygote animals appeared relatively normal within the neocortex at this age (A, B), but NFIX expression was reduced within the periventricular zone of the lateral ventricles (C, D) and within the cerebellum (E, F). Scale bar (in F): A–D 50 µm; E, F 100 µm. (TIF) [file pone.0065478.s001.tif]
